# Antibiotic-mediated changes in the fecal microbiome of broiler chickens define the incidence of antibiotic resistance genes

**DOI:** 10.1186/s40168-018-0419-2

**Published:** 2018-02-13

**Authors:** Wenguang Xiong, Yulin Wang, Yongxue Sun, Liping Ma, Qinglin Zeng, Xiaotao Jiang, Andong Li, Zhenling Zeng, Tong Zhang

**Affiliations:** 1 National Laboratory of Safety Evaluation (Environmental Assessment) of Veterinary Drugs (SCAU) and Guangdong Provincial Key Laboratory of Veterinary Pharmaceutics Development and Safety Evaluation, South China Agricultural University, 483 Wushan Road, Guangzhou, 510642 China; 2 Environmental Biotechnology Laboratory, The University of Hong Kong, Pokfulam Road, Hong Kong, China

**Keywords:** Metagenome, Antibiotic resistance genes, Resistome, Bacterial community, Feces, Chickens

## Abstract

**Background:**

Antimicrobial agents have been widely used in animal farms to prevent and treat animal diseases and to promote growth. Antimicrobial agents may change the bacterial community and enhance the resistome in animal feces. We used metagenome-wide analysis to investigate the changes in bacterial community, variations in antibiotic resistance genes (ARGs), and their bacterial hosts in the feces of broiler chickens over a full-treatment course of chlortetracycline at low and therapeutic dose levels.

**Results:**

The effects of chlortetracycline on resistome were dependent on the specific ARG subtypes and not simply the overall community-level ARGs. Therapeutic dose of chlortetracycline promoted the abundance of tetracycline resistance genes (*tet*A and *tet*W) and inhibited multidrug resistance genes (*mdt*A, *mdt*C, *mdt*K, *omp*R, and *Tol*C). The therapeutic dose of chlortetracycline led to loss of *Proteobacteria* mainly due to the decrease of *Escherichia*/*Shigella* (from 72 to 58%). Inhibition of *Escherichia* by chlortetracycline was the primary reason for the decrease of genes resistant to multiple drugs in the therapeutic dose group. The ARG host *Bifidobacterium* were enriched due to *tet*W harbored by *Bifidobacterium* under chlortetracycline treatment. *Escherichia* was always the major host for multidrug resistance genes, whereas the primary host was changed from *Escherichia* to *Klebsiella* for aminoglycoside resistance genes with the treatment of therapeutic dose of chlortetracycline.

**Conclusions:**

We provided the first metagenomic insights into antibiotic-mediated alteration of ARG-harboring bacterial hosts at community-wide level in chicken feces. These results indicated that the changes in the structure of antibiotic-induced feces microbial communities accompany changes in the abundance of bacterial hosts carrying specific ARGs in the feces microbiota. These findings will help to optimize therapeutic schemes for the effective treatment of antibiotic resistant pathogens in poultry farms.

**Graphical abstract:**

Resistome variations in faecal microbiome of chickens exposed to chlortetracycline

**Electronic supplementary material:**

The online version of this article (10.1186/s40168-018-0419-2) contains supplementary material, which is available to authorized users.

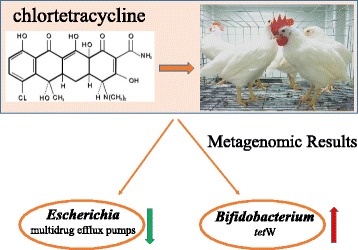

## Background

Antibiotic resistance is one of the most serious global threats to human health, as strongly evidenced by the serious implications of the recent worldwide emergence of KPC-2 (*Klebsiella pneumoniae* carbapenemase-2), NDM-1 (New Delhi metallo-β-lactamase-1), and MCR-1 (plasmid-mediated colistin resistance) [1–4]. Animal fecal microflora harbors a vast reservoir of antibiotic resistance genes (ARGs) that could be acquired by human commensals and pathogens [5]. The antibiotic residues, resistant bacteria, and ARGs in animal excretion may be transported into the environment via manure application, leakage, runoff, and airborne particulate matter [6–8], globally contributing to the aggregation of resistance in the environment.

Poultry production is one of the fastest growing industries worldwide. The global population will reach 8.5 billion by 2030 [9], posing high pressure on international poultry production including the world’s largest poultry producer, China [10]. Large-scale intensive farming systems depend on antimicrobials to prevent and treat animal disease and to enhance growth performance. It is estimated that the global consumption of antimicrobials used for chickens, pigs, and cattle will increase by 67%, from 63,151 tons in 2010 to 105,596 tons in 2030 [11].

Much of the current data regarding the gastrointestinal microbiome in poultry is centered on bacterial communities associated with poultry growth performance and disease status, thus having implications for human health. For examples, Oakley et al. (2014) investigated the effects of formic acid, propionic acid, and medium-chain fatty acids as feed additives on pathogens and found that these treatments did not affect specific pathogens or the cecal microbiome composition [12]. Costa et al. (2017) investigated the impact of antibiotic growth promoters such as zinc bacitracin, enramycin, halquinol, virginiamycin, and avilamycin on the cecal microbiota of broiler chicken and found specific changes in cecal microbiota membership regarding antibiotic growth performance [13]. Wei et al. (2013) examined the effects of supplemental bacitracin and litter management on food-borne enteric pathogens in broiler chickens [14].

Antibiotic administration has profound effects on indigenous microbes of animal feces, leading to changes in microbial community structure and resistance. Chlortetracycline is one of the most commonly used antimicrobials in poultry and swine production [15]. China is the world’s largest producer and consumer of chlortetracycline for veterinary use [16]. However, the changes in microbial community and bacterial resistome in the poultry gastrointestinal tract and resultant feces following chlortetracycline treatment remain largely unknown.

Recently, phages were shown to contribute to antibiotic resistance [17], but they rarely encoded ARGs [18]. Horizontal gene transfer (HGT) via mobile genetic elements such as plasmids, integrases, and transposases are important drivers of ARG dissemination. However, the bacterial community structure rather than HGT has been observed to be the primary determinant of the ARG content in soils [19]. Bacterial community shifts were strongly correlated with the occurrence of antibiotic resistance alterations during drinking water chlorination [20]. The relationship between ARGs and bacterial communities has not been characterized in the feces of animals such as poultry. The identification of bacterial hosts harboring ARGs in poultry feces is helpful for us to optimize a therapeutic scheme to avoid the spread and aggregation of antibiotic resistance.

ARGs can be transported into the environment and acquired by human microbiota via poultry feces used as organic fertilizers for vegetables and fruits [21]. A deep quantitative understanding of the distribution and diversity of ARGs and their bacterial hosts in poultry feces is necessary to evaluate their co-occurrence while they are discharged into the environment and acquired by human pathogens. ARG abundance and diversity were underestimated by previous studies based on cultured bacteria and identified ARGs [22], which is emphasized by increase of sequence-novel ARGs [23]. Sequence-based metagenomic approaches allow a comprehensive exploration of uncultivable and rare taxa from the immense diversity in the fecal microbial population. Recently, although metagenomic approaches have been used to analyze the bacterial communities [24, 25] and ARG abundance in poultry feces [26–28], the responses of ARG-harboring bacterial hosts to antibiotic treatments have been neglected. Chlortetracycline is one of the most commonly used antimicrobials in poultry production for disease prevention and treatment. These diseases include infectious synovitis and air sacculitis that are treated for 3 to 5 days as one course of treatment. Chlortetracycline is also routinely and extensively used in feed for weight gain/feed efficiency in the absence of disease signs and symptoms [29].

We hypothesize that the changes in the structure of antibiotic-induced fecal microbial communities accompany changes in the abundance of bacterial hosts carrying specific ARGs in chicken fecal microbiota. To test this hypothesis, we investigated the impact of a 5-day full-treatment course of chlortetracycline administration on the fecal resistome and microbiota of chickens. By applying 16S rRNA amplicon sequencing and de novo assembly of metagenomic data set, we profiled the changes in fecal bacterial community, the variations in ARG, and bacterial host abundance to demonstrate that antibiotic-induced changes in the abundance of bacterial hosts shape ARG abundance in chicken feces.

## Methods

### Chicken and chlortetracycline exposure

Fifty-four 20-day-old chickens were purchased in the same batch from a commercial production (Guangdong Wens Dahuanong Biotechnology Co., Ltd., China). From days 15 to 22, microbiota maturation occurs and remains in a stable status [25]. We therefore chose 20-day-old chickens for our experimental procedures. At the commercial production, these chickens were raised in cages under standard commercial conditions and had not been treated by any antimicrobials. The chickens were transported to Laboratory Animal Center of South China Agricultural University and divided into three groups (see Additional file 1: Figure S1). Each group had three replicates. Each replicate had six chickens per cage with equal representations of body weight and sex. The chickens were raised in cages and fed under standard commercial production conditions and received a standard commercial corn-soy diet (Yellow little chicken feed, Guangdong Wens Dahuanong Biotechnology Co., Ltd., China) and water ad libitum. The chickens were raised for 7 days before chlortetracycline administration. A 5-day course of chlortetracycline was administered at 2 g/L (the therapeutic dose group) and 0.2 g/L (the low-dose group) in the drinking water. One group without chlortetracycline administration was set as the control group. Each group had three cages, and each cage contained six chickens. Feces from every cage were cleaned daily. Mixed fresh feces from six chickens in each cage were collected on T0 (day 0, before treatment), T5 (day 5, during treatment), and ST10 and ST20 (day 10 and 20, respectively, during stopped treatment) (see Additional file 1: Figure S1). The fresh feces were collected into sterilized tubes using a sterilized spoon, and the sterilized tube was put into an aseptic plastic bag. Three biological replicates were conducted in the treatment and control groups. All the samples were kept on ice and were immediately transferred to laboratory for processing. Chlortetracycline administration was performed from 8 August, 2015, to 28 August, 2015. No clinical symptoms or signs such as diarrhea, allergy, and other disease outbreaks were observed in these chickens during the experiment.

### DNA extraction

Fresh feces from six chickens in one cage were mixed as one replicate. DNA was extracted from three biological replicates using MoBio PowerSoil DNA isolation kit (MoBio Laboratories, Carlsbad, CA, USA) according to the Human Microbiome Project protocol. DNA concentrations and purity were measured by UV spectroscopy using a NanoDrop ND-2000 instrument (NanoDrop Technologies, Wilmington, DE, USA).

### 16S rRNA gene profile

Sequencing of 16S rRNA gene was performed using Illumina MiSeq with a paired-end 300-bp sequencing strategy. The 16S primers (319F and 806R) were used to amplify the hypervariable V3-V4 region of bacterial 16S rRNA genes [30]. Primers also included Illumina sequencing adapter, pad, linker, and the reverse primer contained a 12-bp error-correcting barcode [31]. PCR was performed using a Bio-Rad thermal cycler Model C1000 (Bio-Rad, Richmond, CA, USA). The reaction mixture (50 μL) consisted of 1 μL of DNA template, 0.5 μL of each primer (50 pmol), 5 μL of 10× *Ex* Taq buffer containing 20 mM Mg^2+^, 0.25 μL of *Ex* Taq DNA polymerase, and 5 μL of dNTP mixture (2.5 mM). The PCR procedure was as follows: initial 94 °C denaturation for 3 min, followed by 30 cycles consisting of denaturation (94 °C for 30 s), annealing (50 °C for 30 s), extension (72 °C for 30 s), and a final extension step (72 °C for 10 min). No-template controls were included as negative controls. Clean reads were obtained using the mother MiSeq standard operating procedure (http://www.mothur.org/wiki/MiSeq_SOP) [31]. Briefly, 16S rRNA gene reads were denoised by using pre.cluster, and chimeric sequences were checked by Uchime [31]. On average, we obtained 33,123 clean reads for each sample (Additional file 2: Table S1). We then picked operational taxonomic units (OTU) at 97% sequence identity using the QIIME analysis pipeline [32]. Taxonomic assignments were classified at 80% bootstrap confidence using the Ribosome Database Project [33]. For comparisons across groups, the sequencing depth of samples was normalized to 18,888 reads.

### Metagenomic sequencing

The DNA from fecal samples for 16S rRNA gene sequencing was simultaneously used for metagenomic sequencing. Metagenomic sequencing was performed using Illumina Hiseq 4000 with the sequencing strategy of Index 150 PE (paired-end sequencing). Low-quality reads were filtered to ensure that (1) the reads were aligned to proper adaptors or primers, (2) the reads contained < 10% unknown bases, and (3) the reads contained > 50% high-quality bases [34]. On average, 8.7 Gb clean reads were generated for each sample.

### Calculations of ARG abundance

ARG abundance was determined using ARGs-OAP [35]. Briefly, potential ARG reads and 16S rRNA genes were extracted, and ARG-like reads were identified and annotated using BLASTX by applying the combined ARG database of CARD (The Comprehensive Antibiotic Resistance Database) and ResFinder (freely accessed on 8 September, 2017). Reads were annotated as ARG-like reads at the cutoff of *E* value of 10^− 7^, sequence identity of 90% and alignment length more than 25 amino acids. This identification approach had been validated to 99.5% accuracy by our group [28, 36]. The abundance of ARG-like reads was calculated and normalized by the number of 16S rRNA genes (defined as relative abundance). ARG abundance was expressed as copies of ARGs per copy of 16S rRNA gene in order to be directly compared with the results acquired using qPCR [28]. ARG types and subtypes were automatically counted by using a package of customized scripts previously described by our group [36].

### Metagenome assembly

Clean reads in three replicates for each treatment were de novo assembled with default *k*-mer size using the CLC Genomics Workbench (Version 6.0.2, CLC Bio, Aarhus, Denmark). We obtained 148,151 contigs with average length of 1690 bp (Additional file 2: Table S2).

### Identification of ARG-like ORFs

Open reading frames (ORFs) within contigs were predicted using Prodigal v.2.60 [37]. ORF coverage was calculated by mapping metagenomic reads to the contigs with a minimum length coverage of 95 at 95% similarity [27]. ARG-like ORFs were determined using BLASTX against the combined ARG database mentioned above at *E* value ≤ 10^− 10^ [38] with a minimum similarity of 80% over 70% of query coverage [27]. Coverage (times per Giga base, ×/Gb) of ARG-like ORFs were defined as follows:

$$ \mathrm{Coverage}\kern0.5em =\kern0.5em \sum \limits_1^n\frac{N\kern0.5em \times \kern0.5em 150/L}{S} $$

where *N* is the number of reads mapped to ARG-like ORFs, *L* is the sequence length of target ARG-like ORFs, *n* is the number of ARG-like ORFs, 150 is the length of Illumina sequencing reads, and *S* is the sequencing data size (Gb) [27].

### Taxonomic assignment of bacterial hosts of ARGs

Protein sequences of ORFs predicted in the contigs that carried ARG-like ORFs were annotated using BLASTP against NCBI NR database (downloaded on 2 February, 2015) at *E* value ≤ 10^− 5^. The BLASTP results were annotated using MEGAN (MEtaGenome ANalyzer, Version 5) [39] for assignment of taxonomic genus. The contig was assigned to the taxon if the voting score was more than 50% [11, 40].

### Statistical analysis

Statistical comparisons were done using nonparametric Kruskal-Wallis tests. A *P* value of < 0.05 was regarded as statistically significant. Hierarchical clustering was conducted using the similarity index of Bray-Curtis by the algorithm of unweighted pair-group method with arithmetic averages.

### Submission of sequence data

All sequence data have been deposited in the NCBI Sequence Read Archive (accession no. SRP091637 for metagenomic analysis and accession no. SRP091694 for 16S analysis).

## Results

### ARG variations

On average, we found 5.1 ARG copies per 16S rRNA gene calculated from all fecal metagenomes of broiler chickens (Additional file 2: Table S3). The predominant ARG types were multidrug resistance genes (46%), aminoglycoside resistance genes (10%), and tetracycline resistance genes (6.5%) (Additional file 1: Figure S2). Metagenomic analysis showed that the ARGs were diverse and abundant in feces of broiler chickens, even when chlortetracycline was not administered in control animals. In these control groups, the relative abundances of total ARGs ranged from 4.3 to 6.0. Chlortetracycline treatment at low or therapeutic doses did not significantly alter the relative abundance of total ARGs, nor did it result in increases in the three predominant ARG types (Additional file 1: Figure S2).

We also found that genes resistant to multidrugs and tetracyclines occurred predominantly via drug transporters (Additional file 2: Table S4). For example, the multidrug resistance genes *mdtE*, *mdt*L, *mdt*P, *mdt*N, and *mdt*F were in the 20 most abundant ARG subtypes. Interestingly, *tet*A was the most abundant ARG subtype. In contrast, most aminoglycoside and β-lactam resistance genes uncovered ARGs with antibiotic-inactivating capabilities, via covalent modification of aminoglycosides (*aad*A and *aad*E) and enzymatic degradation of β-lactams (class C β-lactamase). The quinolone resistance gene *qnr*S was primarily plasmid associated. Importantly, *mcr-1* gene occurred as high frequencies. This gene was first reported by our laboratory as a plasmid-mediated polymyxin resistance gene [3]. We located the *mcr-1* gene on a novel contig (Additional file 1: Figure S3).

**Fig. 1:**
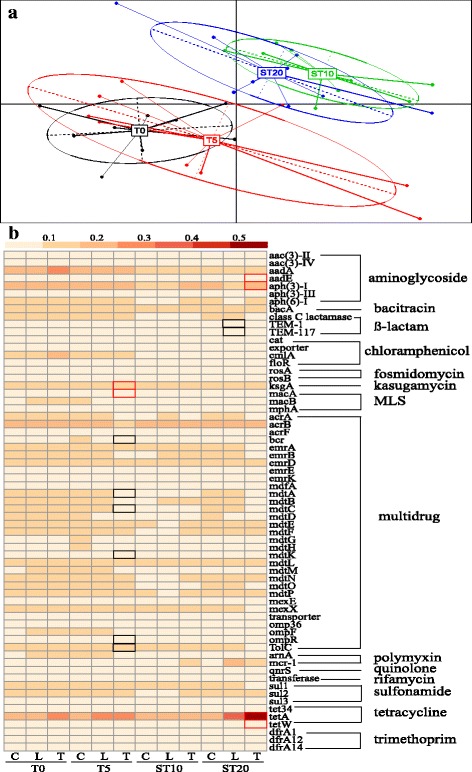
Variations of ARG subtypes in different samples over the course of chlortetracycline administration. **a** Principal component analysis of ARG subtypes. **b** Heatmap of variations of ARG subtypes based on the relative abundance of ARG subtypes. Significant (*P* < 0.05) increases and decreases are shown by border lines of cells colored by red and black, respectively. ARG subtypes with an average abundance > 1% are shown. See Additional file 2: Table S4 for the relative abundances of ARG subtypes

We further analyzed the ARG subtype variations to quantitatively compare the effects of chlortetracycline on the fecal bacterial resistome. The shift of samples as illustrated by principal component analysis (PCA) suggested an impact of chlortetracycline on the fecal resistome at the level of ARG subtypes over time (Fig. 1a). The tetracycline resistance genes (*tet*A and *tet*W) significantly increased in dose-dependent manner in the low-dose and therapeutic dose treatment groups on T5 (*P* < 0.05). In contrast, some ARG subtypes significantly decreased due to chlortetracycline treatment (on T5), such as several multidrug resistance genes (*mdt*A, *mdt*C, *mdt*K, *omp*R, and *Tol*C) (Fig. 1b and Additional file 2: Table S4).

### Changes in bacterial community structure

We analyzed bacterial community structure using sequence data of 16S rRNA genes. We found that *Proteobacteria* (81%), *Firmicutes* (11%), *Bacteroidetes* (2.7%), and *Actinobacteria* (1.3%) were the predominant taxonomic phyla in fecal microbiota of broiler chickens (Additional file 1: Figure S4 and Additional file 2: Table S5). These four main taxonomic phyla also represented the predominant human fecal microbiota [41]. Our data contrasts with a previous study that found *Firmicutes* (56%), *Bacteroidetes* (36%), and *Proteobacteria* (1.7%) from chicken feces using the same sequencing approach [42].

**Fig. 2:**
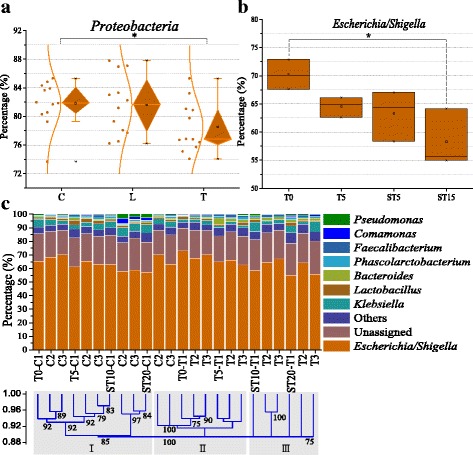
Changes in bacterial community structure. **a** Changes in taxonomic *Proteobacteria* over time among the different groups. **b** Changes in taxonomic *Escherichia/Shigella* over time in the therapeutic dose group. **c** Changes in taxonomic genera in the therapeutic dose group compared with the control group. Others in Fig. 2c mean the percentage of each taxonomic genus < 0.5%. Details of the percentage of taxonomic phyla and genera are shown in Additional file 2: Tables S5 and S6, respectively

There were no significant compositional shifts of the predominant *Proteobacteria* and *Firmicutes* in the low-dose group compared with the control group over time (*n* = 27). This suggested that the structure of the fecal taxonomic phyla was relatively stable over time, even after 5 days of low-dose chlortetracycline treatment. However, the therapeutic dose of chlortetracycline led to significant shifts in the composition of taxonomic phyla including the loss of *Proteobacteria* (from 83% on T0 to 75% on T5) compared with the control group (*P* = 0.045, *n* = 27) (Fig. 2a).

As the predominant genus of *Proteobacteria*, *Escherichia*/*Shigella* significantly decreased from 70% on T0 to 58% on ST20 in the therapeutic dose group (*P* = 0.028, *n* = 12) (Fig. 2b and Additional file 2: Table S6). In contrast, there was no significant shift in *Escherichia*/*Shigella* in the control group (*P* = 0.16, *n* = 12). The decrease of *Escherichia*/*Shigella* mediated by chlortetracycline was the major contributor to the loss of taxonomic phylum of *Proteobacteria* in the therapeutic dose group observed above. The overall depletion of *Escherichia* in the community structure corresponded to the decrease of ARG-harboring *Escherichia*. In addition, the therapeutic dose level of chlortetracycline shifted the fecal microbiome into a distinct structure of taxonomic genera (Fig. 2c) and was most likely due to the broad-spectrum activity of chlortetracycline against both Gram-positive and Gram-negative bacteria. The taxonomic genera in the control and the therapeutic dose groups were clustered into three classes. In the control group, most samples clustered into class I. In the therapeutic dose group, the samples on T0 and T5 clustered into class II, and the samples on ST10 and ST20 clustered into class III. This indicated that the therapeutic dose of chlortetracycline resulted in the divergent changes in taxonomic genera. The divergent changes were also supported by PCA, in which PC 1 accounted for 80% of the variations between samples that were spatially separated from each group (Additional file 1: Figure S5).

### Changes in bacterial hosts of ARGs

The current study achieved a greater depth of sequencing than previous studies of fecal metagenomes of poultry [27, 43], permitting an unparalleled contig assemble and comparison of ARGs. We obtained 2.08 × 10^12^ clean reads that were assembled into 1,777,809 contigs containing 4,105,873 ORFs with an average length of 646 bp (Additional file 2: Table S2). We annotated 4812 ORFs as ARG-like ORFs that were located in 4443 contigs. To quantitatively compare resistomes and their bacterial hosts at higher resolution, we taxonomically assigned these ARG-carrying contigs and normalized the coverage of ARG-like ORFs.

**Fig. 3:**
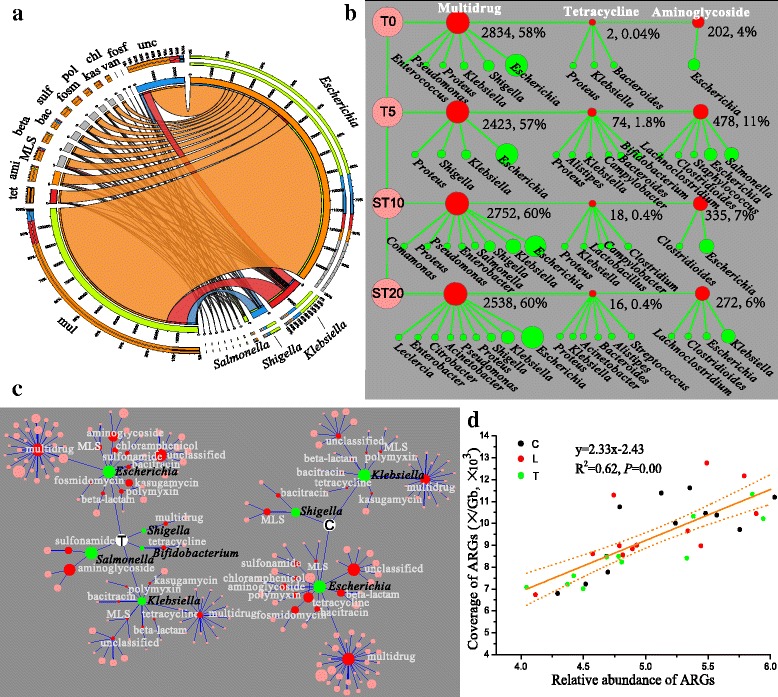
ARGs and their bacterial hosts. **a** Percentages of ARG-carrying bacterial hosts. *Mul* multidrug, *tet* tetracycline, *ami* aminoglycoside, *MLS* macrolide-lincosamide-streptogramin, *beta* beta-lactam, *bac* bacitracin, *sul* sulfonamide, *fosm* fosmidomycin, *pol* polymyxin, *kas* kasugamycin, *chl* chloramphenicol, *van* vancomycin, *fosf* fosfomycin, *unc* unclassified. Details are shown in Additional file 1: Figure S6 and Additional file 2: Table S7. **b** Variations of the predominant ARGs carried by bacterial hosts over time in the therapeutic dose group. ARGs and bacterial hosts are colored by red and blue nodes, respectively. Node size corresponds to percentage. ARGs and their bacterial hosts are connected by line. Details are described in Additional file 2: Table S8. The percentage of a single ARG was calculated using the coverage of the ARG divided by the sum of the coverage of all ARGs in one sample. **c** Changes of bacterial hosts carrying ARGs from the control group compared to the therapeutic dose group on T5. *C* the control group, *T* the therapeutic dose group. Green nodes, red nodes, and pink nodes mean hosts, ARG types, and ARG subtypes, respectively. Node size corresponds to percentage. Bacterial hosts with a percentage of > 1% are shown. Details are described in Additional file 2: Table S9. **d** Linear relationship between the relative abundance of total ARGs and the coverage of total ARGs harbored by bacterial hosts. Each dot represents one sample, and a solid line means a linear fit. The dashed lines represent 95% confidence intervals

ARGs were harbored by diverse genera including *Escherichia*, *Klebsiella*, *Shigella*, and *Salmonella* (Fig. 3a and Additional file 1: Figure S6), suggesting that the fecal resistome resided in phylogenetically diverse microbiota. *Escherichia* was the predominant bacterial host (83%) and harbored most of diverse ARGs including the genes resistant to multidrug, tetracycline, aminoglycoside, macrolide lincosamide streptogramin, β-lactam, and sulfonamides. In a study of dairy cow manure, ARGs originated from *Proteobacteria*, *Firmicutes*, *Bacteroidetes*, and *Actinobacteria* [44]. More than half of genes resistant to multidrug (85%), tetracycline (95%), aminoglycoside (80%), macrolide lincosamide streptogramin (76%), and β-lactam (79%) resided in *Escherichia*, whereas vancomycin resistance genes mainly distributed in *Enterococcus* (100%) and fosfomycin resistance genes mainly distributed in *Klebsiella* (53%) and *Enterobacter* (47%).

The changes in the three most abundant ARG types significantly influenced the overall fecal bacterial resistome (Fig. 3b). During the experimental duration, *Escherichia* was always the major host for the multidrug resistance genes, while the major host for aminoglycoside resistance genes was *Escherichia* on T0, but changed to *Klebsiella* on ST20. The coverage of the multidrug resistance genes decreased up to 15% with antibiotic treatment on T5 and did not recover to the initial level on T0 at the end of the experiment on ST20 (Fig. 3b). Inhibition of *Escherichia* by chlortetracycline was the primary reason for the decrease of multidrug resistance genes in the therapeutic dose group. The coverage of *Escherichia* that harbored multidrug resistance genes decreased by 13 to 17% on T5, ST10, and ST20 compared with T0. This was the primary contribution to the decrease of the hosts harboring these genes (Additional file 1: Figure S7 and Additional file 2: Table S10).

The enrichment of tetracycline resistance genes was due to the emergence of a new set of bacterial hosts that included *Bifidobacterium* (1.3%) on T5 (Fig. 3c). ARG hosts *Bifidobacterium* were enriched after chlortetracycline treatment in the therapeutic group compared to the control group. These ARG contents harbored by *Bifidobacterium* partially explained the enrichment of ARG-harboring hosts. We found that *Bifidobacterium* harbored *tet*W, and not surprisingly, the hosts *Bifidobacterium* were enriched under chlortetracycline treatment.

We further analyzed the correlation between the abundance of total ARGs (calculated by raw reads) and the coverage of total ARGs (based on metagenomic assembly) that were harbored by bacterial hosts in each sample. We found a significantly positive linear relationship (*y* = 2.33 × − 2.43, *R*^2^ = 0.62, *P* = 0.00), even treated by the low and therapeutic dose of chlortetracycline (red and green dots in Fig. 3d). These results clearly showed that the ARGs harbored by bacterial hosts were dictated by the total ARG reads, and it was, therefore, predictable, even with chlortetracycline treatment. The good linear relationship suggested that assembled ARGs can represent raw read ARGs, and our analysis of ARG hosts based on the contig assembly is appropriate.

## Discussion

Numerous studies of antibiotic resistance using traditional bacterial culture methods have provided information concerning existing and emerging antibiotic resistance threats [4, 45]. Over time, the study of antibiotic resistance has grown from single cultured microorganisms to exploring antibiotic resistance in diversified bacteria including pathogenic, commensal, and environmental bacteria at the level of microbial communities [46]. In the current study, we here adopted a high-throughput approach, next-generation sequencing, to analyze the effects of a broad-spectrum antibiotic on the fecal resistome and bacterial communities in chickens, highlighting the antibiotic-induced alterations of ARG-harboring bacterial hosts in microbial communities.

The most common ARG types including the genes resistant to multidrug, aminoglycoside, and tetracycline detected in this study were also found in other specific niches, such as human feces and the environment [28, 47, 48]. However, the predominant ARGs found in this study were different from soil in which β-lactamases were the most frequently encountered [19] and were also different from human feces, river water, and sediments [28]. This supported that ARGs are not randomly distributed in diverse environments. We found an average of 5 ARG copies per 16S rRNA gene in both the control and chlortetracycline treatment groups. This level of ARG abundance was greater than that observed in feces from commercial chicken flocks (3.1 copies) because of updated ARG databases [28]. The ARG levels from chickens with different backgrounds of antibiotic use indicated that the ARGs might be established in poultry fecal metagenomes in the absence of antibiotic exposure. Similarly, ARGs were established in human feces within the first week after birth independent of antibiotic treatment [49]. Regardless of antibiotic selection, early establishment of fecal bacterial resistome most likely originated from the environmental microbiota. For instance, the existence of ARGs in Hadza hunter-gatherers with little antibiotic exposure supported the ubiquitous presence of environmentally derived resistances [50], which are the weapons that environmental microorganisms use to compete against other microbes. We found that chlortetracycline treatment did not significantly increase the relative abundance of total ARGs, nor did it alter the three predominant ARG types. This suggests that the effects of antibiotics on the fecal bacterial resistome were dependent on ARG subtypes and not simply on the overall community level of ARG types.

The therapeutic dose of chlortetracycline caused the loss of *Proteobacteria* (from 83 to 75%), which was mainly contributed by the decrease of *Escherichia*/*Shigella* (*P* = 0.028). Human fecal microbiome yet did not resemble to the pre-treatment status, even more than 1 year after antibiotic treatment [51]. The extent of recovery to the pre-treatment levels depends on diverse factors including microbiota structure, nature of antibiotic, course of treatment, and host genetics [52]. In the human study, two individuals responded to ciprofloxacin with a similar decrease of *Proteobacteria*, but these were distinct from variations of *Actinobacteria*, *Bacteroidetes*, *Firmicutes*, and *Verrucomicrobia* [53]. Alterations in fecal taxonomic phyla depend on class and number of courses of antibiotics [54], as well as diet [55–57], age [58], geographic location [59], and disease status [60].

Tracking the dynamics of ARG-harboring bacterial hosts under antibiotic exposure at the community level is central to understanding the emergence of resistance in microbial ecosystems. We determined the distribution of ARG-harboring bacterial hosts at the taxonomic genus level and tracked the effects of chlortetracycline treatment. The ARG-harboring genera at the community-wide level indicated that the fecal resistome resided in phylogenetically diverse microbiota such as *Escherichia*, *Klebsiella*, *Shigella*, and *Salmonella*. We also found that same ARG type was present in different genera but had preferences. For example, more than half of the genes resistant to multidrug (85%), tetracycline (95%), aminoglycoside (80%), macrolide lincosamide streptogramin (76%), and β-lactam (79%) resided in *Escherichia* while vancomycin resistance genes mainly distributed in *Enterococcus* (100%). These results supplement our understanding of the distribution of ARGs and their bacterial hosts in animal fecal metagenomes and provide precise administration strategy to treat resistant pathogens. For example, *Enterococcus* was the predominant bacterial host resistant to vancomycin, so we should treat *Enterococcus* infection using the effective antimicrobials that *Enterococcus* was sensitive to. We provide a novel insight into precision medicine through specific targeted administration of antimicrobials against resistant bacteria.

Antibiotic-alteration of bacterial community structure significantly influenced ARG-harboring bacterial hosts. We found that inhibition of *Escherichia* by a therapeutic dose of chlortetracycline as revealed by the bacterial community structure was the primary reason for the decrease of multidrug resistance genes. In addition, the enrichment of *Bifidobacterium* imposed by a therapeutic dose of chlortetracycline was due to the tetracycline resistance gene *tet*W they harbored. The antibacterial activity of chlortetracycline selected for resistant bacteria and inhibited the sensitive bacteria. This caused shifts in bacterial abundances that in turn changed resistome structure in the fecal metagenome. The concomitant ARG alterations and their bacterial hosts suggested the presence of widespread association between the fecal resistome and the bacterial community structure. Our findings suggested that antibiotic-mediated alteration of bacterial community structure shapes ARG contents in fecal microbiota of chickens under chlortetracycline treatment.

## Conclusions

We conducted an integrated analysis of changes in microbiome structure and variations of ARG contents to obtain a comprehensive view of the fecal ecosystem dynamics following antibiotic treatment. The effects of chlortetracycline on the fecal resistome were dependent on the specific ARG subtypes and not simply overall ARGs in the community level. The changes in the structure of the antibiotic-induced fecal microbial community accompany changes in the abundance of bacterial hosts carrying specific ARGs. When the conclusions were adapted in other studies, many factors should be considered, including antibiotic spectrum, duration, and delivery route, as well as antibiotic susceptibility, microbial community structure, and contig assembly.

## Additional files

Additional file 1: **Figure S1.** Animal groups and sampling time points. **Figure S2.** ARG types in chicken fecal metagenomes. **Figure S3.** Comparisons of the genetic environment of *mcr-1*. **Figure S4.** Changes in taxonomic phyla over time in different samples. **Figure S5.** Principal component analysis of taxonomic genera in the therapeutic dose group and in the control group. **Figure S6.** ARGs and their bacterial hosts. **Figure S7.** Changes in dominated bacterial hosts carrying ARGs in the therapeutic group. (DOCX 1230 kb)

Additional file 2: **Table S1.** Information on sequencing depth of 16S rRNA genes. **Table S2.** Information on assembly, contigs, and ORFs. **Table S3.** Relative abundance of ARG types. **Table S4.** Relative abundance of ARG subtypes and their significance in different groups. **Table S5.** Changes in taxonomic phyla over time in different samples (%). **Table S6.** Changes in taxonomic genera over time in the therapeutic dose group and in the control group (%). **Table S7.** Average percentages of bacterial hosts carrying antibiotic resistance genes (%). **Table S8.** Variations of predominant ARGs carried by bacterial hosts over time in the therapeutic group. **Table S9.** Details of bacterial hosts and their harboring ARGs in the control group and in the therapeutic dose group on T5. **Table S10.** Changes in predominant bacterial hosts carrying ARGs in the therapeutic group. (XLSX 120 kb)

## Abbreviations

ARGs: Antibiotic resistance genes
HGT: Horizontal gene transfer
OTU: Operational taxonomic units
