# Supplementary material for: Antibiotic-mediated changes in the fecal microbiome of broiler chickens define the incidence of antibiotic resistance genes
Source: Microbiome. 2018 Feb 13;6:34. doi: 10.1186/s40168-018-0419-2 (PMC5811963; doi:10.1186/s40168-018-0419-2)
Supplement: Supplementary file 1 — Figure S1. Animal groups and sampling time points. Figure S2. ARG types in chicken fecal metagenomes. Figure S3. Comparisons of the genetic environment of mcr-1. Figure S4. Changes in taxonomic phyla over time in different samples. Figure S5. Principal component analysis of taxonomic genera in the therapeutic dose group and in the control group. Figure S6. ARGs and their bacterial hosts. Figure S7. Changes in dominated bacterial hosts carrying ARGs in the therapeutic group. (DOCX 1230 kb) [file 40168_2018_419_MOESM1_ESM.docx]

**Supporting Information-Figures**

**Contents**

**Figure S1.** Animal groups and sampling time points…………………………………………2

**Figure S2.** ARG types in chicken faecal metagenomes…………………...………….………3

**Figure S3**. Comparisons of genetic environment of *mcr-1*…………………………...………4

**Figure S4.** Changes in taxonomic phyla over time in different samples…….……………….5

**Figure S5.** Principal component analysis of taxonomic genera in the therapeutic dose group and in the control group…………………………………………………….……….………..6

**Figure S6.** ARGs and their bacterial hosts……………………………………………………7

**Figure S7.** Changes in dominated bacterial hosts carrying ARGs in the therapeutic group….8

**References**……………………………………………………………………………………9


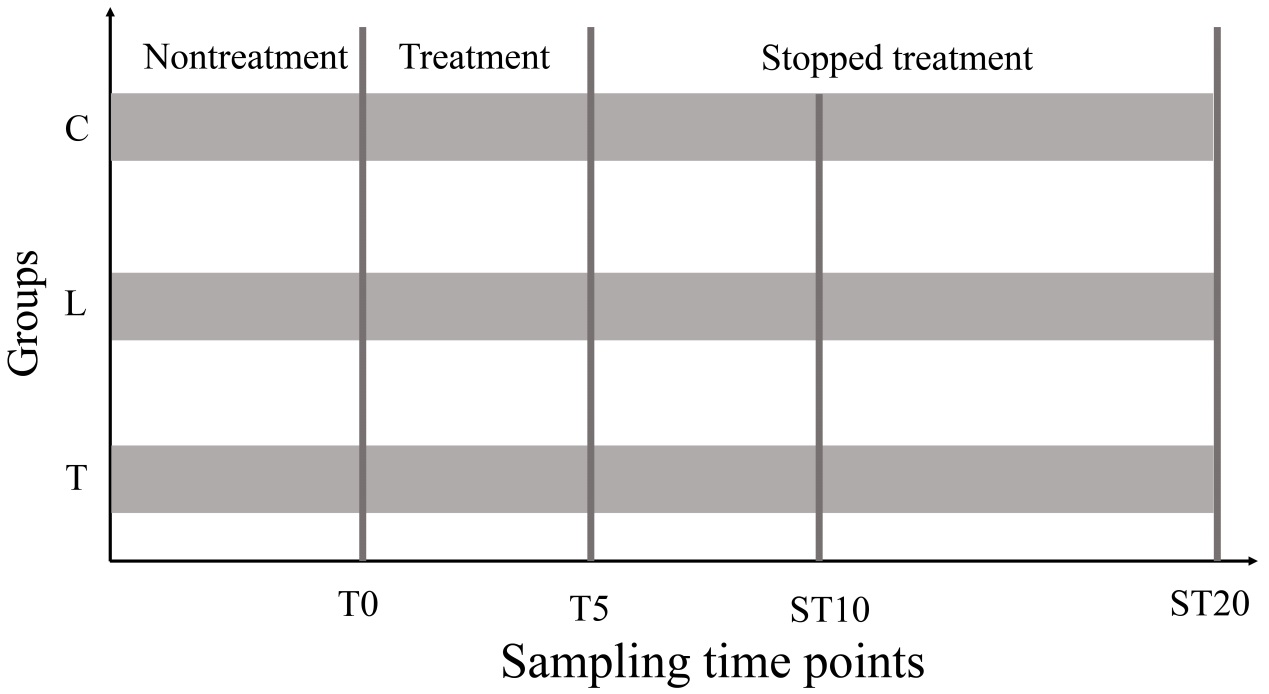


**Figure S1.** Animal groups and sampling time points. The 54 broiler chickens were divided into three groups. Each group had three replicates. Each replicate had six broiler chickens in one cage, with equal representation of body weight and sex. A 5-day course of chlortetracycline was administered at 2 g/L (the therapeutic dose group, T) and 0.2 g/L (the low dose group, L). One group without chlortetracycline administration was set as the control group (C). Fresh feces from each group were collected on T0 (day 0, before treatment), T5 (day 5, during treatment), ST10 and ST20 (day 10 and 20, respectively, during stopped treatment).


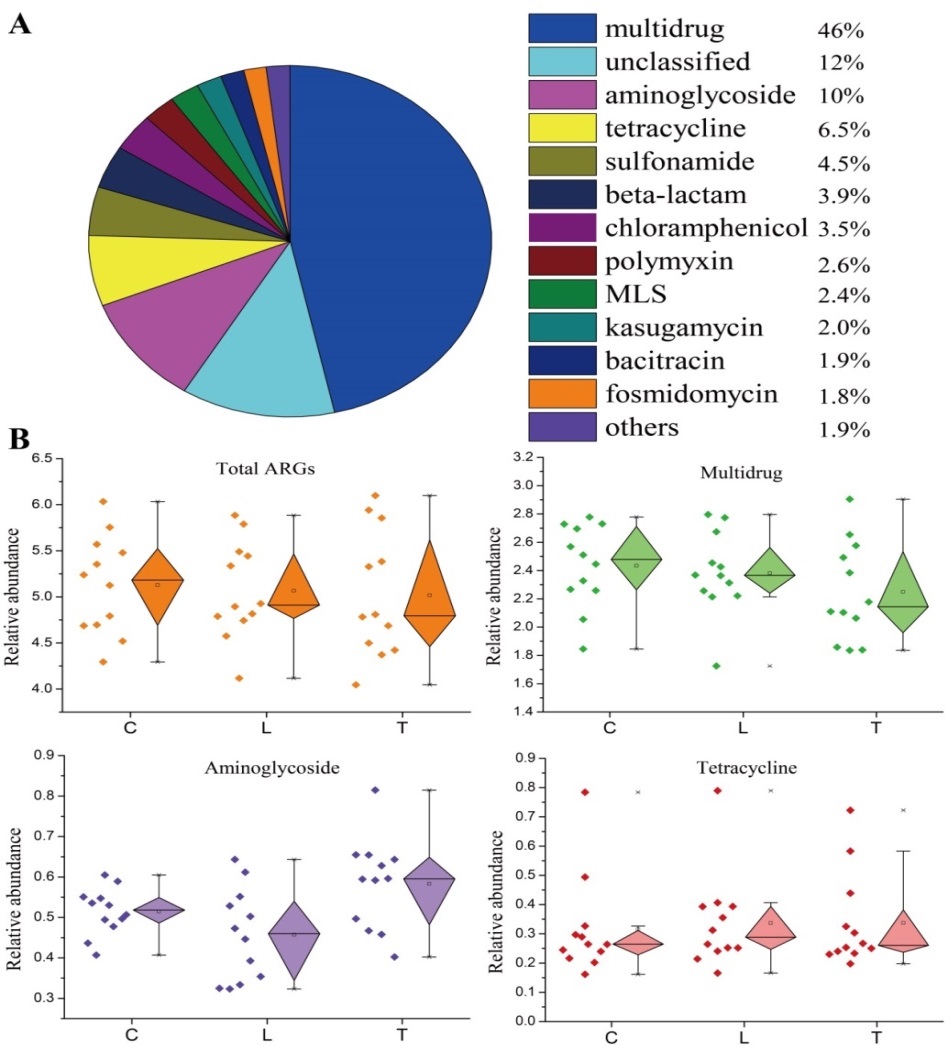


**Figure S2.** ARG types in chicken faecal metagenomes. (A) Percentages of all ARG types. (B) Variations of relative abundance of ARG types in different groups. The pie chart was drawn based on average abundances of ARG types (ARG copies/16S rRNA gene copies) shown in **Table S3**. The box plot diagram was displayed with the 25^th^ and 75^th^ percentiles shown by a box, the median shown by a hollow point, and the minimum and maximum shown by whiskers. No statistical significance of relative abundance of ARGs was observed among the three groups in each box plot.


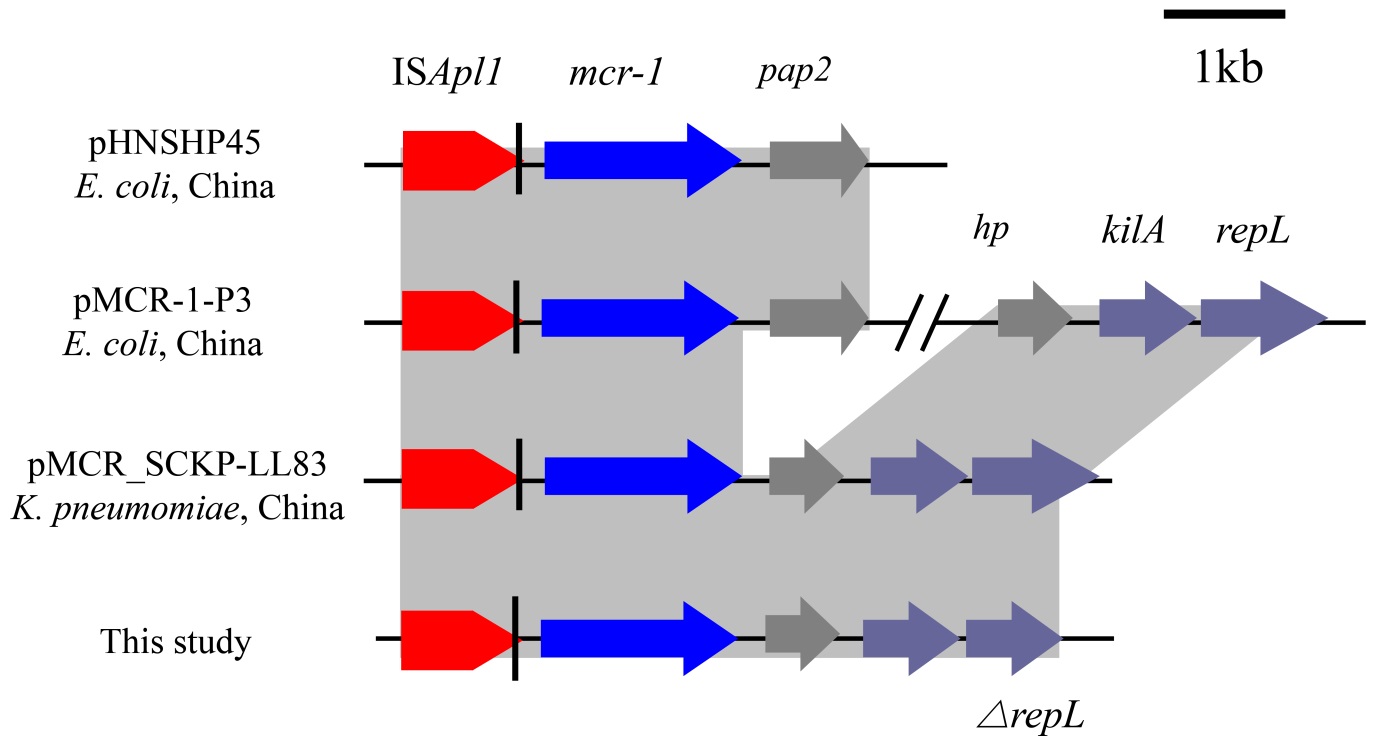


**Figure S3.** Comparisons of genetic environment of *mcr-1*. pHNSHP45 and pMCR-1-P3 have been published [1, 2], while pMCR_SCKP-LL83 just was deposited to NCBI without publication (on 20 October, 2017). The genetic environment of *mcr-1* found in this study was similar to the sequence structure of pMCR_SCKP-LL83.


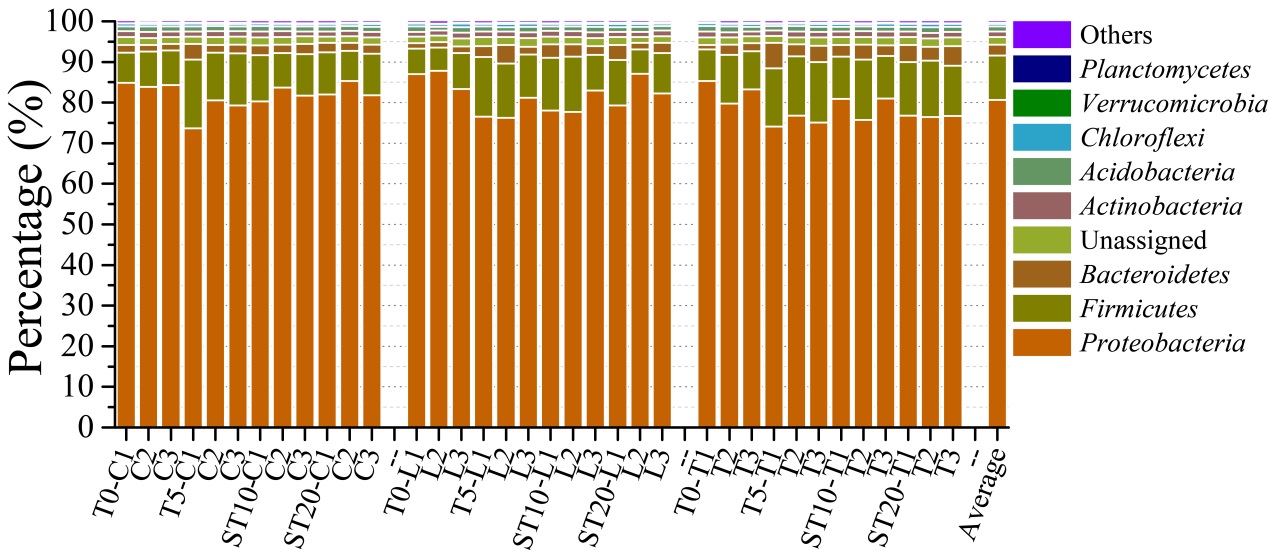


**Figure S4.** Changes in taxonomic phyla over time in different samples. Others mean the percentage of each taxonomic phylum less than 0.1%. Details of the percentage of taxonomic phyla and genera are shown in **Table S5**.


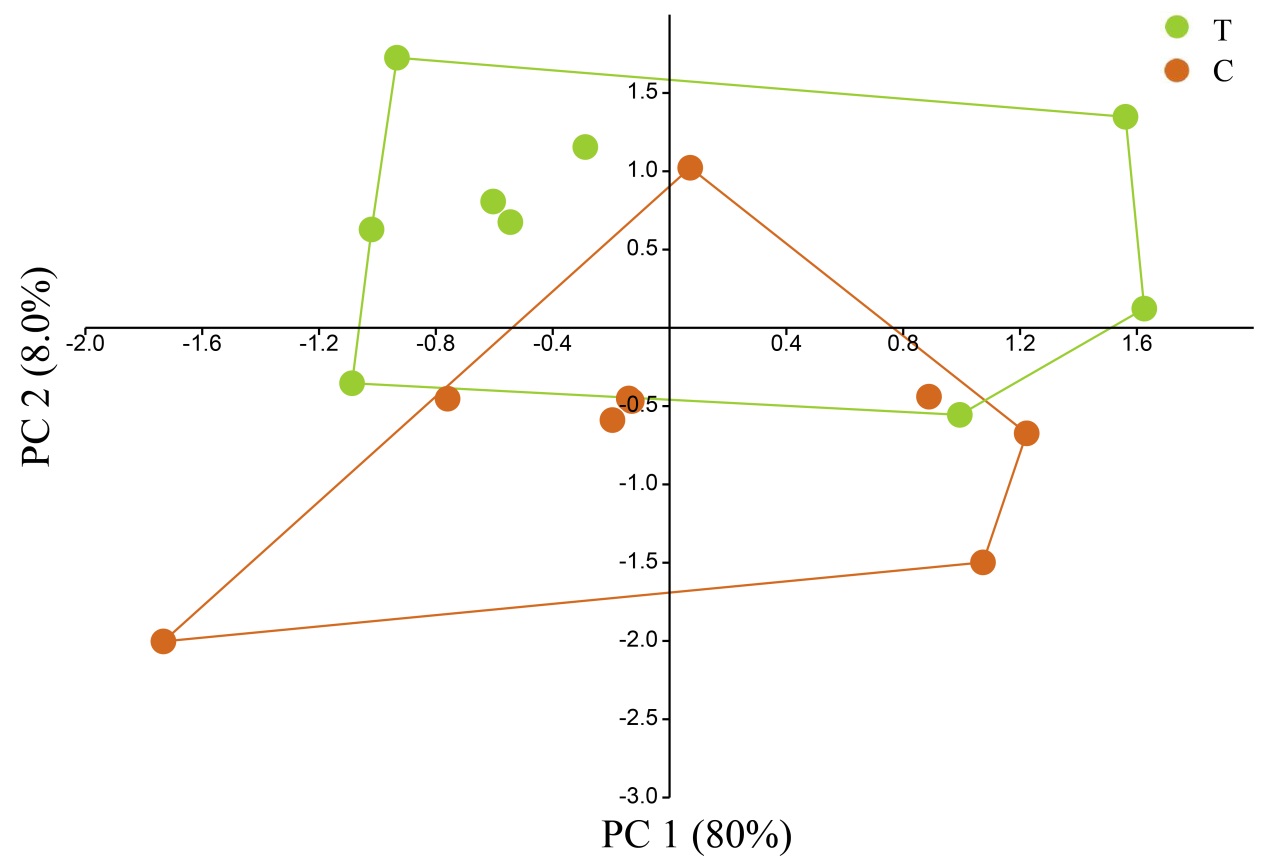


**Figure S5.** Principal component analysis of taxonomic genera in the therapeutic dose group and in the control group. T, the therapeutic dose group. C, the control group. Samples collected on T0 in both groups were excluded in the figure, since they were not treated by chlortetracycline on T0.


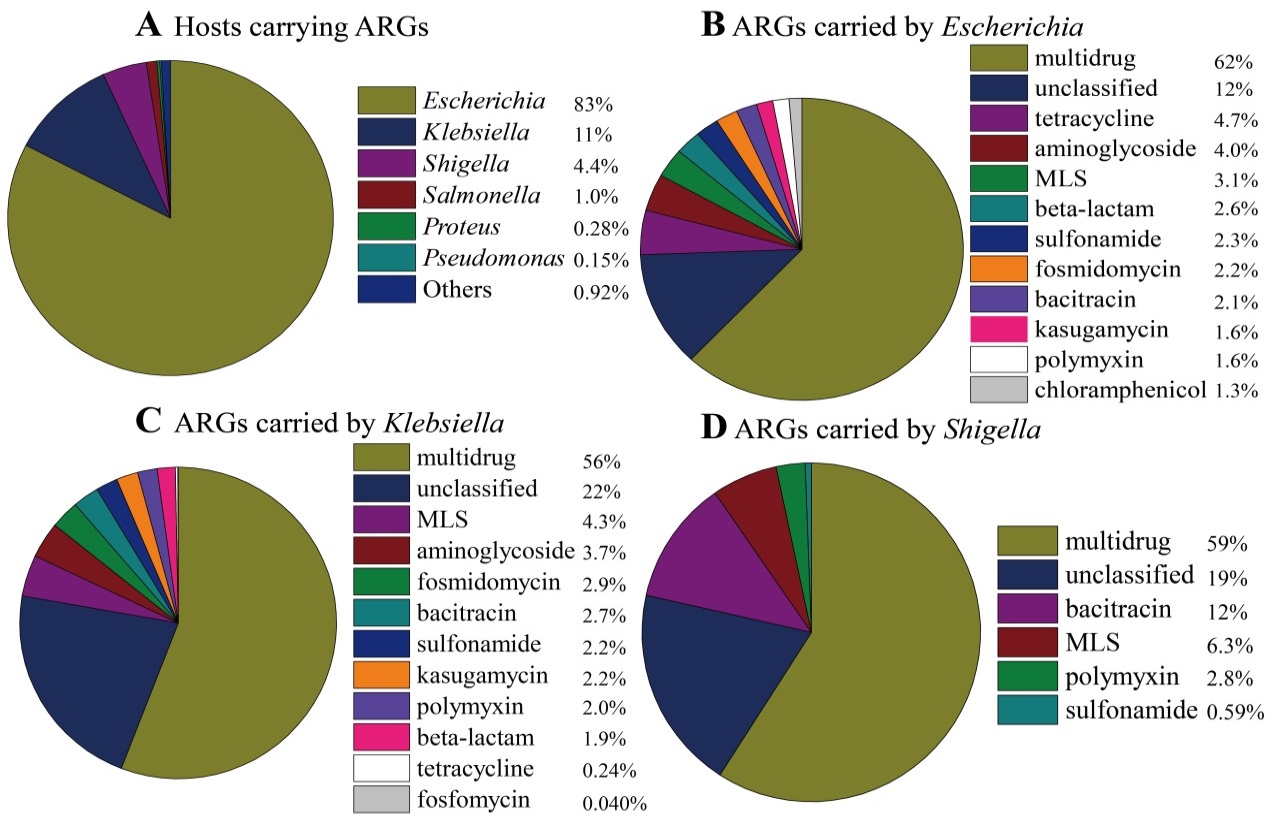


**Figure S6.** ARGs and their bacterial hosts. (A) The percentage of bacterial hosts carrying ARGs. The distribution of ARGs carried by main hosts including *Escherichia* (B), *Klebsiella* (C) and *Shigella* (D). Others mean that the percentage of each bacterial host was less than 0.1%.


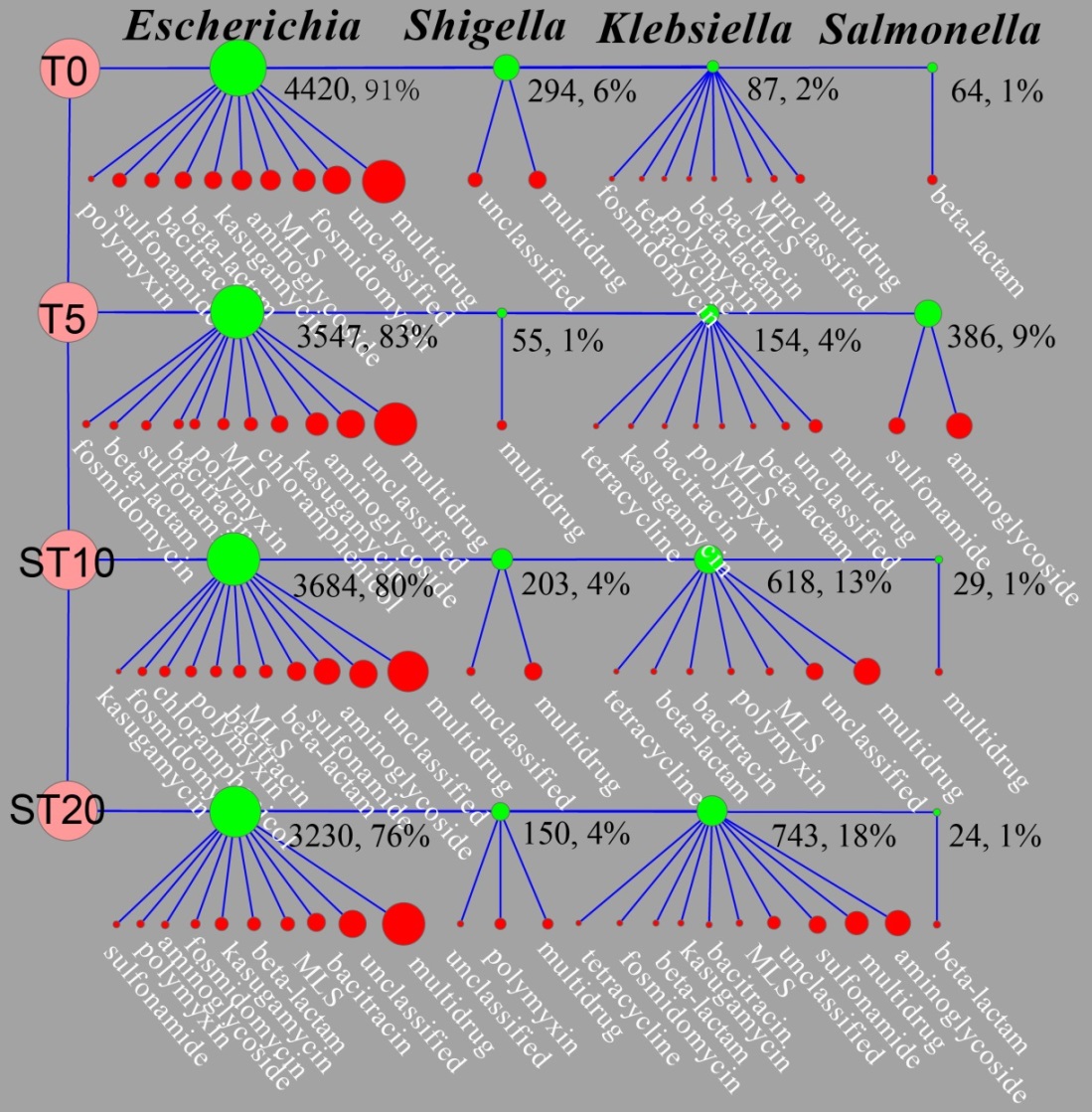


**Figure S7.** Changes in dominated bacterial hosts carrying ARGs in the therapeutic group. Hosts and ARGs are colored by blue and red, respectively. ARGs and their bacterial hosts are connected by line. Coverage (×/G) and percentage (%) of each host are shown. Details of bacterial hosts and their harboring ARGs are described in **Table S10**.

**References**

[1] Liu Y-Y, Wang Y, Walsh TR, Yi L-X, Zhang R, Spencer J, et al. Emergence of plasmid-mediated colistin resistance mechanism MCR-1 in animals and human beings in China: a microbiological and molecular biological study. The Lancet infectious diseases 2016;16(2):161-8.

[2] Zhang C, Feng Y, Liu F, Jiang H, Qu Z, Lei M, et al. A phage-like IncY plasmid carrying the *mcr-1* gene in Escherichia coli from a pig farm in China. Antimicrobial agents and chemotherapy 2017;61(3):e02035-16
